# A taboo within a stigma? a qualitative study of managing incontinence with people with dementia living at home

**DOI:** 10.1186/1471-2318-11-75

**Published:** 2011-11-14

**Authors:** Vari M Drennan, Laura Cole, Steve Iliffe

**Affiliations:** 1 Faculty of Health & Social Care Sciences, Kingston University and St. George's University of London, UK; 2 Research Department of Primary Care and Population Health, University College London Medical School, UK

## Abstract

**Background:**

Incontinence in people with dementia is one of the factors associated with the decision to move to a care home. Managing incontinence adds to carer burden and has been reported by family carers as more difficult to manage than behavioural symptoms. Active management strategies have been reported to be associated with less carer depression. The purpose of this study was to investigate carers' perceptions of the range of incontinence problems they helped their relative with and the strategies they employed to manage these.

**Methods:**

Family carers of people with dementia living in their own homes were recruited through primary care, specialist community mental health services and voluntary organisations. Qualitative semi structured interviews were conducted either face to face or by telephone and thematically analysed.

**Results:**

Thirty two carers were interviewed. They described a range of problems from supporting the person to remain independent in toileting, through to dealing with inappropriate behaviours, to containing and managing incontinence. All carers actively used problem solving strategies but sometimes these were not acceptable or understood by the person with dementia, particularly as the dementia progressed. Most carers reported protecting the person's dignity by not seeking health professionals help often until the point of a crisis. Once the carer has decided to seek help the responses from health professionals can be less than helpful, and carers report local health service policies on access to continence products to be inconsistent and often inappropriate to their circumstances. A few carers reported strategies for managing toileting and incontinence that have the potential for distress and harm to the person with dementia.

**Conclusions:**

Primary care professionals could be more proactive in enquiry, repeated over time, about toileting and incontinence problems and in giving advice and information to reduce crisis and problems.

## Background

Incontinence is reported as the most problematic symptom to manage by family carers of people with dementia [1]. It is a factor which contributes significantly to both family carer 'burden' associated with supporting a person with dementia [2] and also to the decision to seek residence in a group or care home [3]. Family carers of people with dementia have higher levels of stress and depression than those caring for people with other conditions [4]. There is some evidence that carer coping strategies in managing the incontinence contributes to their social isolation for example limiting activities to within the home [5]. In the face of increasing numbers of people with dementia [6,7], health professionals and policy makers in many countries are seeking ways to support family carers and enable people with dementia to remain in their own homes [8,9]; this requires consideration of issues such as support for the management of incontinence.

The clinical syndrome of dementia has a trajectory of progressive deterioration in cognition, abilities in undertaking activities of daily living, and physical functioning [10]. The impairment experienced by the individual is often compounded by extrinsic factors such as attitudes of '*therapeutic nihilism*' (i.e. attitudes of nothing can be done to help) in professionals [11] and environments un-adapted to the needs of people with dementia. Many people also develop behavioural and psychological symptoms (BPSD) [12], which can manifest in inappropriate voiding and toileting behaviours [13]. As the dementia advances it interferes with neurological pathways for controlling voiding resulting in incontinence [14]. The high prevalence of incontinence in people with dementia resident in care homes is well documented [14]. However, international estimates suggest over two thirds of all people with dementia live in their own homes [15,16] but there are no prevalence studies of people with dementia living at home with incontinence although there are some indications. A UK population based study of 15,000 home dwelling people aged over 75 identified that 18.3% had cognitive impairment and of these 31% had urinary incontinence problems [17]. Evidence from Australia suggests 78% (n = 23,000) of co-resident carers to people with dementia provide personal care [18]. Data from a survey of 1,480 American carers reported that of those caring for a person with dementia 32% were providing assistance in toileting and 31% were managing incontinence and pads [19]; although it is not evident if these are the same or different carers.

Incontinence is the involuntary leakage of urine or faeces or both, with the term 'functional' incontinence used to describe that which has no physiological basis [20]. The International Continence Society suggests that for frail elderly people, which includes people with moderate to severe dementia, the aim of professional input may be to have 'well contained' incontinence i.e. through the use of incontinence pads [21]. There is some evidence from a care home population that health professionals, family carers and residents have different views on what is acceptable in continence care [22]. This descriptive, comparative study of preference in the treatment of urinary incontinence between frail older residents of nursing homes, family members and nursing staff, found nurses stated preferences for prompted voiding techniques. In contrast some older people and family members reported such techniques as embarrassing and fostered dependency and so were not preferred [22]. Family carers of people with dementia may be coping with a range of toileting and incontinence problems, however, there is little evidence from their perspective to inform and guide professional input. This study contributes to the evidence base by exploring the perspective of the carers on the range of problems in toileting and incontinence they deal with and the strategies for managing them.

## Methods

The research approach was in the interpretative tradition as appropriate to the question of interest [23]. A topic guide was constructed for semi-structured interviews with family carers of people with dementia living at home either conducted face to face or by telephone as preferred by participants. The interviewer first invited participants to identify any problems they were having or had experienced with toileting or incontinence using any terms and expressions they felt comfortable with. Participants were then asked to talk through their strategies for managing those problems, identifying those that worked and any help or assistance they had in that. The ordering of questions allowed rapport to develop prior to the discussion of potentially very intimate and embarrassing information. Prior consideration was also given as to how to manage both any distress or sadness and also any disclosure that might suggest neglect or harm to a vulnerable adult. A purposive sample was sought to ensure diversity in gender, relationship to the cared for person, ethnicity and socio-economic groups. Recruitment was through multiple sources: general practice, specialist community mental health services and voluntary organisations and continued until saturation of themes was reached [23]. Interviews were taped, with permission, and transcribed. Thematic analysis [24] undertaken with the aid of NViVO software [25] first by reading and coding by individual researchers, then agreeing the overall thematic framework and resolving any disagreement in discussion. A favourable ethical review was received from Camden and Islington NHS Research Ethics Committee and informed consent was obtained from all participants.

## Results

Thirty two carers participated in the study: 21 in face to face interviews and 11 by telephone. There were slightly more women (n = 19) than men (n = 13) and the majority were spouses (n = 21). Although postcode and self-reported ethnicity was not given for all participants there was some diversity in socio-economic area of residence and ethnicity. Ethnicity other than white British included Afro-Caribbean, Asian and European. Table 1 reports the characteristics.

**Table 1:** Characteristic of Participants

*Characteristics*	*Participants*			
*Gender *	19 female	13 male	-	
*Relationship to cared for *	21 spouse	10 adult child	1 daughter in law	
*Living in area as categorised by the Index of Multiple Deprivation(IMD) for postcode *[26]	2 in area of band of 30% most deprived areas by IMD	11 in 40% middle band of areas by IMD	5 in area of band of 30% least deprived areas by IMD -	14 full postcode not obtained
*Ethnic Origin *	12 White British	9 other (Afro-Caribbean, Asian and European)		11 not given in telephone call

The carers described a wide range of problems: some as a result of the loss of cognition and memory, some from behavioural and psychological symptoms and some from the interplay of these with other co morbidities, as well as over medication such as the person with dementia self medicating with laxatives unbeknown to the carer. The toileting and incontinence problems they described included:

• The inability to act in a timely way to the sensation of the need to void,

• The inability to locate, recognise and use toilets; as well as undo locks on toilet doors once inside,

• The inability to manage the personal activities of toileting i.e. undoing and replacing clothing, for men how to direct the penis, and how to undertake subsequent cleaning and personal hygiene,

• Inappropriate management of soiled clothing as well as urine and faeces e.g. hiding soiled underwear, attempting to hand faeces to the carer, wiping faeces off hands onto clothing or furniture,

• Voiding in situ i.e. where the person was standing or sitting without any attempt to remove clothes or move to an appropriate place,

• Choosing to void in an inappropriate place e.g. defecating in the garden, standing by the bed at night, or in inappropriate receptacles e.g. waste bins,

• Resistance to others helping with toileting.

### Carer developed strategies and issues of acceptability

Most carers described problem solving by themselves, or with immediate family, without seeking advice or help from professional sources. For many this was an active choice as they sought to protect the person's dignity.

"*I didn't want people to know about her not managing and incontinence. Not for what I did [cleaning her after voiding], I felt her dignity, you know. I felt I didn't want anybody to know. She was so in herself a very dignified lady, you know, and to suddenly be like that. If she'd have known she'd have been horrified, and I didn't want people to know that either" Husband*

Carers described a trajectory of initially acting in ways to prevent problems such as reminding the person to go to the toilet on a regular basis, in one instance a daughter doing this by telephone from work, to then moving to more active assistance in the acts of toileting and then seeking out and obtaining aids and absorbent products. The carers problem solving strategies mirrored the trajectory of the dementia and required them to consider strategies for day and night time, inside and outside the home. This trajectory of problem solving strategies was similar for all participants, irrespective of relationship or other characteristic. One noticeable difference however was that those resident in more wealthy areas described the purchase of aids such as commodes and different types of incontinence pads in response to new problems while those living in areas of higher socio-economic deprivation described a reliance on access to National Health Service (NHS) provision of aids and incontinence pads.

Three different issues were identified with regard to the acceptability of the solutions. The first was the acceptability of prompts and aids. While many carers reported to successfully using prompting and reminding to go to the toilet at an early stage in incontinence and memory problems, a few reported that prompting or reminding led to irritation and arguments as the person with dementia perceived this as being treated like a child. Only a few carers reported using location and orientation reminders such as pictures of toilets on walls and doors to help direct the person to the bathroom. Similarly these were reported as unacceptable to some people with dementia, e.g. '*offended her sense of propriety*', who removed them. Some family carers reported buying or obtaining commodes for night time use which were either not recognised as a 'toilet' or were viewed as unacceptable objects outside a bathroom by the person with dementia.

The second issue of acceptability raised was whether such intimate assistance was acceptable to the person with dementia. As the dementia progressed, the family carers described taking a more active role in helping the person with dementia to locate the toilet, remove clothing, use the toilet, attend to intimate personal hygiene after voiding, replace clothing and then wash hands. Some family carers described the embarrassment of their relative in requiring such help. They reported trying to minimise the embarrassment by giving reassurance and using humour. Some family carers described more violent responses to help with toileting from people with greater cognitive impairment, indicating a lack of acceptability for whatever reason:

"The problem is she doesn't like people touching her. She knows she wants to go to the toilet, you get her there, then she doesn't want to pull her trousers down, so you have to start, you have to do it, so she's going to fight......If she gets your fingers she'll try and break them ". ...Daughter

The third issue related to gender and the acceptability of men helping women in the intimate act of toileting. Husbands described helping wives in toileting but those that had sought help with the problem had some public provision of home carers visiting the home to help with toileting. This did not always seem to be apparent for wives assisting husbands. The sample was too small to identify if there were particular influences such as availability of local services, socio-economic factors or ethnicity. A linked but separate issue was whether it was seen as acceptable for sons to assist mothers in the intimate act of toileting. One example of a strategy to avoid sons helping a mother with dementia in this type of care was a daughter -in-law who had taken on the role of assisting her mother-in-law, including sleeping at her mother-in-law's home to help at night. There was only one participant who was a son of a mother with dementia who described how within the family it was seen as unacceptable for him to help:

"Well mum needs help to go to the toilet; my sister won't let me keep company with her on my own. ...She just says I can't help mum, it's not right and I can't be the one to be there in an evening and give her a break. We've had the most god awful rows". Son

### Seeking help from health professionals

Some carers described how the extent of their problems with toileting and incontinence was only revealed to health professionals as part of a crisis event such as admission to hospital. Others reported that the admission to hospital or respite care had iatrogenic consequences with the person previously continent but discharged incontinent and using pads

So we are managing, he is dry because I help him and then he has to go into hospital and that's it - 7 days and he comes back out using pads. No one has even tried to help him - bam! Straight into pads. It takes me weeks to get him to use the toilet again". Wife

Many of those, who described seeking advice from general practitioners in non crisis situations, reported that they felt the responses unhelpful either from the brevity of response or because they seemed not to take full cognizance of their situation or the dementia.

"When she got constipated, the GP gave me suppositories. Even though my mother was even-tempered and trusted me, trying to insert them was a battle because she couldn't understand what I was up to ...as you can imagine." Daughter

While some carers could point to health care professionals who had both helped in the management of the problems and also acknowledged their emotional response to the situation, the majority recounted lengthy periods of trying and failing to get appropriate help and advice to their situation.

A common narrative was that when they approached a health professional for help that person a) failed to understand the impact and consequences of the problem(s) and b) referred or directed them to another professional or service who similarly was unable to help.

"*My most dreadful day was Boxing Day when my mother emerged from the lavatory carrying a pile of faeces which she dumped on the little social services trolley she ate from. After this I managed to get a visit from the consultant geriatric psychiatrist, a copy of whose letter the GP sent me. It says something about the daughter expressing concerns about hygiene but no help or advice." Daughter*

### Consequences for carers and the need to contain excreta

Carers described multiple impacts managing these problems had. Most described an emotional response to dealing with the personal hygiene of another adult, particularly after defecation: both distaste and then normalisation '*just getting on with it'*. For a few, the distaste and the feelings of contamination remained dominant:

"I found the smell and trying to get rid of them [used continence pads] most distressing. I got to where I would take the rubbish bags with the soiled pads to the shared bins in the middle of the night so I wouldn't meet any of the neighbours". Wife

The financial consequences were described in terms of the additional laundry costs, the replacement of soiled furniture, floor covering and mattresses, as well as the purchase of cleaning materials, room sprays to mask smells, furniture and mattress protection and incontinence pads.

All the carers reported that an important management strategy was that of containment of urine and faeces in absorbent pads- at first for what were described as 'occasional accidents' but then for ongoing problems in toileting and incontinence. They reported problems in finding incontinence pads that were acceptable to the person with dementia (both when unused and soiled) and effective in containing the excreta. While some reported that pad designs that most closely resembled underpants, i.e. 'pull-up pants' were often most acceptable to the mobile person with dementia, others reported different designs were more suitable to their relatives. All reported difficulties in finding advice, information about these products and ways of identifying the most suitable without purchasing multiple boxes. While some reported excellent support from district nurses in the continence assessment and then supply of National Health Service (NHS) funded continence pads, others re-counted a process they described as inadequate or unmanageable for a person with dementia and their carer.

"Even the district nurses and the incontinence service seemed unable to provide the kind of insight into managing incontinence in patients with dementia which I required." Wife

Most also reported that their local NHS services had criteria about the provision of continence pads to community dwelling patients that restricted access to both the designs and also quantity of products which they considered they needed to manage. Most described buying additional products in response to perceived NHS restrictions and failures of the delivery processes. The reported purchasing of different types of incontinence products was least evident in those living in the most socio-economic deprived areas.

It was evident that carers were choosing strategies that minimised the impact of the problem upon themselves as well as the person with dementia. A common strategy reported to prevent nocturia was the restriction of fluids later in the day. The use of incontinence pads to reduce the strain on both the carer and the person with dementia getting up in the night was commonly reported.

And this business of being wet at night....... I thought I would avoid that by getting her up a couple of times during the night to use the commode'... but it's the whole palaver of getting her out onto the commode, having to wait. ...And so I thought 'well, let's go on the internet and find out which pants will absorb the most liquid' (laughs). Daughter in law, resident for part of the week (8)

It was not always clear whose best interest the strategy had been chosen for and the carer had to confront the difficult task of weighing up the use of an effective strategy with the potentially perceived negative outcome of using such a strategy (i.e. the person with dementia's human rights) in the face of limited alternatives.

"So there were faeces everywhere it was just disgusting, it was terrible...So now we put the belt on his trousers but we put the buckle right at the back.... he can't get to it. So when he wants to go, he has to go in the pad. So when we come in, no mess. I racked my brains but I couldn't think of anything else to do." Daughter

In one instance a distressed carer shared that both he and paid carers used considerable force to insist the person with dementia sat on the toilet. With his permission we relayed this information and his distress to the clinical team who had first introduced us.

## Discussion

This study offers two new insights that will help practitioners make better decisions about helping with continence management in community dwelling people with dementia. The reluctance to seek help by carers of person with dementia as they seek to preserve the dignity and personhood [11] has not been reported before, although the low rate of seeking help for incontinence symptoms is well documented in older adults [26]. Similarly, the differing perspectives on the acceptability or otherwise of strategies to prevent incontinence have been observed before in a care home setting [22] but not in community dwelling populations.

This is a relatively small qualitative study, conducted in an English setting, and as such has limitations. However, the diversity of the sample in gender, relationship to the person with dementia, ethnicity and socio-economic circumstances combined with recruitment of participants until saturation in data categories suggests that a breadth of carer experience has been captured. The volunteer recruitment process through multiple organisations and agencies means there is no information on those who did not volunteer to participate. The size of the sample meant the influence and interplay of factors such as gender, relationship to the person with dementia, ethnicity, and socio-economic circumstances on incontinence problem solving decisions both by carers and those around them, both family and professionals, could not be explored in depth and requires further investigation.

Management strategies are mediated by the degree of acceptability of the solutions to both the person with the dementia, the carer and other family members. Carers also judge the feasibility and effectiveness of management strategies through the assessment of the impact on themselves both physically and emotionally. Decision making in this context leads to favouring the use of incontinence pads rather than assisting the person to use the toilet. As in the International Continence Society guidance [21], the carers overarching aim is for 'well contained' incontinence. The distress and impact of managing urine and faeces that is not well contained is identified here as a key influence in carers management strategies. This raises the question as to whether poorly contained incontinence, rather than incontinence itself, is the significant factor influencing decisions changing residence to a care home [2,3]. This requires further investigation.

This study also confirms and provides more detail about the range of problems carers experience in managing the toileting and incontinence needs of a person with dementia at home [5,13]. It confirms the reliance on prompted voiding and pads [27] but details the difficulties that carers have in finding appropriate information and advice in using both these techniques. The evidence here suggests that continence related problems change over time and that repeated clinician and practitioner led enquires become important. These propositions require further exploration.

Each country's health care system differs in the extent to which incontinence pads are both funded and/or provided. The evidence here, specific to England, suggests that local provision may not be sufficiently responsive to the needs of people with dementia living at home. In this it lends further support to the recent Royal College of Physicians national audit of continence care which highlighted that "the quality of continence care remains variable and in some respects remains poor"[28].

While carer coping strategies with people with dementia have been well described [29], this study adds to the literature through detailing that some care decisions are made that privilege carer concerns over the person with dementia preferences and may be counterproductive or even harmful. Clinicians and practitioners should remain alert to the possibility that this type of decision making could be counter to the best interests of the person with dementia. Clinicians and practitioners need to enquire about the strategies and offer alternatives. Primary care health professionals may need knowledge of a wider repertoire of strategies. This too requires further exploration.

## Conclusion

The geriatric giant of incontinence [30], as experienced by people with dementia at home and carers, remains untamed. Clinicians and practitioners, particularly those in primary care could be more proactive in enquiry, repeated over time, about toileting and incontinence problems. This will allow counter-productive or harmful strategies to be identified and alternatives offered. More openness about incontinence may assist carers to discuss the problems they are having managing it, as the taboo of incontinence inside the stigma of dementia is reduced. Professional responses can be poor, and services inadequate. There is a need for enhancing the management skills of doctors and nurses who work with people with dementia, so that they can help with incontinence management. Clinicians and commissioners who are attentive to patient feedback may need to take urgent action about incontinence services that are inappropriate and unresponsive.

## Competing interests

The authors declare that they have no competing interests.

## Authors' contributions

VMD designed the study, was co-applicant in obtaining NIHR Programme grant funding, undertook data collection, analysis and interpretation, drafted and revised the paper. LC undertook data collection, analysis and revised the draft paper. SI designed the study, obtained NIHR funding as principal investigator for the NIHR programme grant, undertook analysis and interpretation, and revised the draft paper. All authors read and approved the final version of the manuscript.

## Pre-publication history

The pre-publication history for this paper can be accessed here:

http://www.biomedcentral.com/1471-2318/11/75/prepub
